# Supplementary material for: Contribution of Metabolomics to Multiple Sclerosis Diagnosis, Prognosis and Treatment
Source: Int J Mol Sci. 2021 Oct 15;22(20):11112. doi: 10.3390/ijms222011112 (PMC8541167; doi:10.3390/ijms222011112)
Supplement: Supplementary file 1 [file ijms-22-11112-s001.zip › ijms-1402748-supplementary.pdf]

Table S1. Evidence from the Literature. ***Abbreviations***. **25(OH)D:** 25-Hydroxyvitamin D; **ACh:** Acetylcholine; **AD:** Autoimmune Disease; **ADA:** Anti-Drug Antibodies; **AIDS:** Autoimmune Deficiency Syndrome; **ALS:** Amyotrophic Lateral Sclerosis; **Apo:** Apolipoprotein; **AUC:** Area Under the Curve; **BBB:** Blood-Brain Barrier; **BHIB:** B-hydroxyisobutyrate; **C0:** Free Carnitine; **C2:** Acetyl carnitine; **C16Cer:** Ceramide (d18:1;16:0); **C16GlcCer:** Glucosylceramide (d18:1;16:0); **C18Cer:** Ceramide (d18:1/18:0); **CIS:** Clinically Isolated Syndrome; **CJD:** Creutzfeldt-Jakob Disease; **CNS:** Central Nervous System; **CP:** Chronic Progressive; **CSF:** Cerebro-Spinal Fluid; **CVD:** Cerebro-Vascular Disease; **FAs:** Fatty Acids; **GBS:** Guillain-Barré Syndrome; **GC-MS:** Gas-Chromatography Mass-Spectroscopy; **GSH:** glutathione; **GSH-Px:** Glutathione Peroxidase; **GTAs:** Gastrointestinal Tract Acids; **HCA:** Hierarchical Cluster Analysis; **HDL:** High Density Lipoproteins; **HIAA:** Hydroxyindoleacetate; **HPLC:** High Performance Liquid Chromatography; **HRMS:** High Resolution Mass Spectroscopy; **HTP:** Hydroxytryptophan; **IIH:** Idiopathic Intracranial Hypertension; **ITM:** Idiopathic Transverse Myelitis; **KA:** Kynurenic Acid; **KP:** Kynurenine Pathway; **K-PLS-DA:** Kernel Partial Least Square Discriminant Analysis; **KYN:** Kynurenine; **KYNA:** Kynurenate; **LDL:** Large Density Lipoproteins; **LC-MS:** Liquid-Chromatography Mass-Spectroscopy; **LysoPC:** Lysophosphatidylcholines; **MBP:** Myelin Basic Protein; **MRS:** Magnetic Resonance Spectroscopy; **MS:** Multiple Sclerosis; **NBV:** Normalized Brain Volume; **NGMV:** Normalized Grey Matter Volume; **NMR:** Nuclear Magnetic Resonance; **NAA:** N-acetyl aspartate; **NMO:** Neuromyelitis Optica; **NMOSD:** Neuromyelitis Optica Spectrum Disorder; **NO:** Nitric Oxide; **ns:** non-specific; **NWMV:** Normalized White Matter Volume; **OIND:** Other Inflammatory Neurological Diseases; **OND:** Other Neurological Diseases; **OPLS:** Orthogonal Projections to Latent Structure; **PC:** Phosphatidylcholines; **PCA:** Principal Component Analysis; **PD:** Parkinson's Disease; **PDGF-AA:** Platelet Derived Growth Factor AA; **PLA_2**: Phospholipase A^2; **PLS-DA:** Partial Least Square Discriminant Analysis; **PlsEtn:** Plasmalogen Ethanolamines; **PP:** Primary Progressive; **PTX-3:** Pentraxin-3; **QA:** Quinolinic Acid; **ROS:** Reactive Oxygen Species; **RR:** Remitting Relapsing; **SD:** Standard Deviation; **SFA:** Saturated Fatty Acid; **SP:** Secondary Progressive; **TCA:** Tricarboxylic Acid; **TGF-α:** Transforming Growth Factor alpha; **TNF-α:** Tumour Necrosis Factor alpha; **VIP:** Variables Importance in the Projections; **WBC:** White Blood Cell; **VLCFA-PtdEtn:** Very Long Chain Fatty Acids containing Phosphatidyl Ethanolamines; **VLDL:** Very Low Density Lipoproteins.

***These papers combine metabolomics and neuroimaging data to gain insights into pathophysiological mechanisms of MS, to improve diagnosis or to monitor treatment response.**

References	Fluid	No. patients/No. controls	Platform	Findings
**Metabolic differences between MS patients and controls**				
Qureshi et al, 1988	CSF and plasma	12 MS patients/12 controls	LC	• Plasma: ↑ Ala ($p < 0.01$) and ↓ Asp, Met, Val ($p < 0.05$), Lys ($p < 0.01$) in MS patients (disruption of the amino acid transport systems).<br>• CSF: ↑ Arg ($p < 0.05$), Gln, Tyr ($p < 0.01$), and ↓ Asp, Glu, Gly ($p < 0.05$), Phe ($p < 0.01$), Met, GABA ($p < 0.001$) in MS patients (local factor in the brain related to MS condition).
Lynch et al, 1993	CSF	21 definite and 9 probable MS/7 GBS/3 CJD /9 AIDS/5 PD/27 controls	NMR spectroscopy	• Acetate significantly (p=0.032) ↑ and formate significantly (p=0.033) ↓ in MS patients vs controls.<br>• All but two of the spectra from actively progressing MS patients presented a single peak at 2.82 ppm, likely from an N-methyl metabolite, indicating a block in the cholino-glycine cycle**.** This peak was not present in spectra from any of the control subjects.
Koschorek et al, 1993	CSF	29 disk herniations/7 tumours/4 MS/1 borreliosis	NMR spectroscopy	The relative concentrations of valine, alanine, putrescine, glutamine, citrate, glucose and creatinine in MS differed significantly (p value not reported) from those observed in tumours and controls.
Simone et al, 1996	CSF	53 MS (of which 45 had a RR course)/12 idiopathic	NMR spectroscopy	• CSF lactate/creatinine ratio significantly (p=0.036) ↑ in MS patients vs controls only during clinical exacerbation. ↑ mean value of CSF lactate (p=0.017) in MS patients with active vs inactive plaques. Pathological values of CSF lactate (above mean + 2 SD

		polyneuropathies/20 meningitidis/18 controls		in controls, maybe produced by anaerobic glycolytic metabolism of activated leucocytes) detected in 7/13 patients with active plaques but in none of those with chronic plaques.<br>• Relative concentrations of CSF citrate and formate significantly (p=0.05) ↓ in MS patients compared to OIND and controls.
Nicoli et al, 1996	CSF	19 MS /12 degenerative dementia/17 controls	NMR spectroscopy	CSF metabolic profile slightly modified in MS patients vs controls (↑ lactate and fructose, ↓ creatinine and phenylalanine), *p* value not reported.
't Hart et al, 2003	Urine	(*Humans*) 10 MS/20 OND/11 controls (*Non-humans*) 3 ESCUDO/3 9501 monkeys/2 naïve monkeys	NMR spectroscopy	• (*Humans*) Multivariate analysis showed a clear separation between the three clusters of samples. The main differences between MS and non-MS patients were in the non-aromatic region of the spectrum (0.5-4.5 ppm).<br>• (*Non-humans*) Multivariate analysis showed clear separation between the three clusters of samples, mostly due to peaks in the aliphatic region (0.5-3.5 ppm).<br>• Chemical entities in the 0.5 – 3.5 ppm region of the urine spectrum as the most likely candidate markers of inflammatory demyelination.
Sinclair et al, 2009	CSF and serum	(i) Cohort 1: 25 IIH, 12 MS, 9 CVD, 41 others;<br>(ii) Cohort 2: 8 IIH, 3 MS, 14 others	NMR spectroscopy	(i) The PLS-DA models generated from the CSF metabolic profile of IIH and MS patients and the serum metabolic profile of CVD patients predicted diagnosis with good (75-80%) sensitivity and specificity.<br>(ii) In the analysis the 2nd cohort, the CSF metabolic profiles generated from the 1st cohort showed moderate ability to segregate patients with IIH and MS (sensitivity/specificity of 63/75% and 67/75%, respectively).
Mehrpour et al, 2012	Serum	23 MS/28 healthy controls	NMR spectroscopy	• ↑ glucose (p=0.00039) and ↓ valine (p=0.0024) in MS patients vs healthy controls.<br>• Mean selenium level significantly ($p < 0.001$) ↓ in MS patients vs controls.<br>• Low levels of selenium were associated with lower glutamine (glutamine is a precursor to GSH, GSH is a co-factor of GSH-Px and GSH-Px is a selenoprotein).<br>• ↑ selenium was associated with ↑ creatine, which is important in neuroprotection.
Gonzalo et al, 2012	CSF	9 MS /9 non-MS individuals	LC-MS	• MS samples had significantly ($p < 0.05$) ↑ 8-iso-PGF2α (lipid oxidation marker) and ↓ myristic acid and palmitic acid.<br>• Since no increase in lipid peroxidizability or major changes in FAs were observed, ↑ oxidative damage in MS patients may be mainly due to lipoxidation phenomenon (lipid peroxidation in proteins due to ↑ free radical production).<br>• Autoantibodies against lipoxidized proteins, significantly ($p < 0.05$) ↑ in MS, can contribute to disease progression.
Kanceva et al, 2015	Serum	12 MS women/6 controls in the follicular phase of the menstrual cycle	GC-MS	• Significant ↑ of most C21 steroids ($p < 0.05$, perhaps in the attempt to attenuate neuroinflammation), androstenedione (p=0.01) and estriol (p=0.016) in MS patients vs controls.<br>• Most C21 steroids ($p < 0.05$ or $p < 0.01$), two conjugated α/β-reduced androstanes ($p < 0.05$ and $p < 0.01$, respectively) and estriol ($p < 0.05$) positively correlated with the presence of MS in women.
Pieragostino et al, 2015	CSF	13 RR MS /12 OND	LC-MS	• Two lysoPC (p=0.015 and p=0.03, respectively, maybe related to overstimulation of PLA_2), lysophosphatidylinositol (p=0.009), phosphatidylcholine (p=0.037), phosphatidylinositol (p=0.039) and 2 unidentified species at m/z = 523.0 Da (p=0.004) and 523.9 Da (p=0.017) significantly ↑ in MS patients. Phosphatidic acid (p=0.034) and an unidentified signal at 727.0 Da (p=0.022) significantly ↓ in MS patients vs OND subjects. Glutamate (p=0.04) significantly ↑ in MS vs OND patients.<br>• Positive correlation ($p < 0.05$) between the intensity of 2 lysoPC and the unidentified signal at 523.0 Da and the Link index.<br>• Positive correlation ($p < 0.05$) between the intensity of phosphatidylinositol and disease duration (possible role in broadening of neurodegeneration).<br>• Negative correlation ($p < 0.05$) between lysophosphatidylinositol and EDSS (possible protective role against disease progression and severity).
Cocco et al, 2016	Serum	73 MS (61 RR/12 CP)/88 healthy controls	NMR spectroscopy	• Glucose ($p < 0.05$), 5-OH-Trp ($p < 0.001$), Trp ($p < 0.001$) and 3-OH-butyrate ($p < 0.01$) were significantly ↓ in MS vs controls.<br>• Acetoacetate ($p < 0.001$), acetone ($p < 0.001$), alanine ($p < 0.01$) and choline ($p < 0.01$) were significantly ↑ in MS vs controls.
Bhargava et al, 2017	Plasma	*Cross-sectional cohort*:27 RR MS patients/27 controls;<br>*Longitudinal cohort*: 24 RR MS/27 controls	LC-MS<br>GC-MS	• *Cross-sectional cohort*: 43 metabolites differed between MS patients and controls ($p < 0.05$). Using PLS-DA the two groups were clearly separated (p=0.001). Important metabolic pathways driving separation between the groups included redox homeostasis (GSH metabolism and γ-glutamyl amino acids metabolism), urea cycle, histidine and xenobiotic metabolism.<br>• *Longitudinal cohort*: γ-glutamyl-leucine (p=0.01), γ-glutamyl-histidine ($p < 0.0001$), γ-glutamyl isoleucine (p=0.002) and γ-glutamyl valine (p=0.0007) were significantly altered between MS patients and controls over time. After 25(OH)D

				supplementation there were changes in metabolites involved in redox homeostasis, lysolipids and FAs (effect of vitamin D in lowering oxidative stress).
Poddighe et al, 2017	Plasma	32 MS /33 healthy controls	GC-MS	• Phosphate, fructose, myo-inositol, pyroglutamate and threonate were significantly ↓ in MS patients vs controls ($p < 0.05$).<br>• L-asparagine, L-ornithine, L-glutamine and L-glutamate were significantly ↑ in MS patients vs controls ($p < 0.05$).<br>• Performing a pathway analysis, asparagine biosynthesis and citrulline biosynthesis were the most relevant pathways.
Villoslada et al, 2017	Serum	*Retrospective longitudinal cohort*: 238 MS/74 controls; *Prospective cohort*: 61 MS/41 controls	LC-MS	*Prospective cohort*:<br>• Moderate accuracy of PCA (R^2=0.247 and Q^2=0.128) and high accuracy (R^2X=0.414, R^2Y=0.481, Q^2Y=0.348) of PLS-DA in discriminating participants as healthy or MS.<br>• Sphingomyelin and lysophosphatidylethanolamine were the main metabolites of the MS signature ($p < 0.01$).<br>• Both PCA and PLS-DA differentiate relapse-free patients during 2-year follow-up vs patients with relapses and patients remaining with EDSS < 3.0 vs those reaching an EDSS > 4.5.<br>*Retrospective longitudinal cohort*:<br>• Levels of 1-monoacyl-glycerophoaphocholine LysoPC 20:0/0:0 (p=0.0022), LysoPC 20:1/0:0 (p=0.0076), LysoPC 22:5/0:0 (p=0.0116), LysoPC 17:0/0:0 (p=0.0001), arachidonic acid (p=0.0195) and 13-hydroxyoctadecadienoic acid (p=1.94E-09) were associated with the relapse-free status.<br>• Levels of cortisol showed a trend for association with disability (p=0.051).
Rossi et al, 2018	Serum	12 MS women monitored in each trimester (T1/T2/T3) of pregnancy and for three months after giving birth (PP)	LC-MS	• Steroids (except for cortisol and androstenedione in T3) and oestrogens showed a gradual ↑ in each trimester of pregnancy and important ↓ in the post-partum period.<br>• There was a strong difference between the levels of C0 (T1 vs PP p=0.05; T2 vs PP p=0.0001; T3 vs PP p=0.0001), C2 (T1 vs PP p=0.01; T2 vs PP p=0.0001; T3 vs PP p=0.0001), C24 (T1 vs PP p=0.01; T2 vs PP p=ns; T3 vs PP p=0.ns), Gly (T1 vs PP p=0.01; T2 vs PP p=0.0001; T3 vs PP p=ns), Tyr (T1 vs PP p=0.05; T2 vs PP p=0.01; T3 vs PP p=0.05), Pro (T1 vs PP p=0.01; T2 vs PP p=0.05; T3 vs PP p=ns) in T1/T2/T3 and those in the post-partum period.<br>• Ala gradually ↑ from T2 to post-partum, showing a significant difference at T1 vs T3 (p=0.05) and T1 vs PP (p=0.01).<br>• A positive correlation was observed for sphinganine (p=0.01) and ceramides ($p < 0.05$) with progesterone, whereas a negative correlation was found for sphingomyelin d18:0/22:0 (p=0.001), C0 (p=2.01988E-09) and Tyr (p=1.51946E-05) with progesterone.<br>• C16Cer (p=0.029) and C18Cer (p=0.049) showed a significant correlation with EDSS. Beyond oestrogens, other hormonal steroids may have a crucial role in neuroprotection**.**
Andersen et al, 2019	Serum	12 MS patients drug naïve/13 controls	GC-MS	• There was a statistically significant difference between the levels of the following metabolites between MS patients and controls: pyroglutamate (p=0.033), laurate (p=0.035), PC ae C42:5 (p=0.018), tetradecenoyl-L-carnitine (p=0.018), unknown 056 (p=0.048), PC ae C40:5 (p=0.016), N-methylmaleimide (p=0.015) and myoinositol (p=0.034).<br>• HLA-DRB1 (a known MS-related gene) was not associated with any metabolite. The expression of several other HLA genes (HLA-DMA, HLA-DMB, HLA-DOA, HLA-DPA1, HLA-DPB1, HLA-DRA, HLA-DRB3, HLA-DRB6) was associated with acylcarnitine C14:1.<br>• The expression of multiple genes proximal to the 200 putative non-MHC MS risk variants were associated with the metabolites. Especially, KPNB1, CLEC16A, PIK3R2, IKZF1, TXK and PHGDH were associated with at least three metabolites ($p < 0.1$).
Podlecka-Pietowska et al, 2019	CSF	19 MS/19 controls	NMR spectroscopy	• Acetone (p=0.001), choline (p=0.008), urea (p=0.006), 1,3-dimethylurate (p=0.358), creatinine (p=0.007), isoleucine (p=0.140), myo-inositol (p=0.015), leucine (p=0.052) and 3-OH-butyrate (p=0.157) were ↓ in MS vs controls.<br>• Among hydrophobic compounds, –CH_3–saturated, monounsaturated ω–9 and/or ω–7 acyl groups and FA (0.86 ppm) (p=0.003), CH_3–saturated ω-3 acyl groups and FA (0.96 ppm) (p=0.060), –CH_2–CH=CH–acyl groups and FA (1.99 ppm) (p=0.492), –OCO–CH_2–acyl groups in triglyceride (2.25 ppm) (p=0.243), –OCO–H_2–,–COOH– CH^2–acyl groups in 1,3-DG, 1-MG, and FA (2.35 ppm) (p=0.082) and an unassigned signal at 3.33 ppm (p=0.854) were ↓ whereas $ROCH_2$–CHOH–CH_2OH glyceryl group in 1-MG (3.68 ppm) (p=0.107) and =HC–CH_2–CH=diunsaturated ω-6 acyl groups and FA (p=0.861) were ↑ in MS vs controls. This suggests an altered energy metabolism and FA biosynthesis in MS.

De Oliveira et al, 2019	CSF and plasma	13 MS (2 PP /11 MS)/8 OIND/15 IIH	LC-MS	*CSF:*<br>• FAs and polyketides were the main lipids in the inflammatory signature (p=0.086).<br>• Comparing MS and IIH, FAs and glycerophospholipids were the most abundant classes of lipids in the MS signature (p=0.08).<br>• Comparing MS and OIND, FAs and sphingolipids were the most abundant classes of lipids in the MS signature ($p < 0.001$).<br>*Plasma:*<br>• Glycerolipids and FAs were the in the MS signature (p=0.055), suggesting that breakdown of the BBB is as important as the inflammatory process.
Cicalini et al, 2019	Tears and serum	Tears: 12 MS/ 21 controls; Serum: 12 MS/10 controls	LC-MS	• Of 30 putative tear lipid biomarkers, 5 have been already found in the CSF (molecular cross-talk between tears and CSF). ↓ sphingomyelins in MS were the most relevant lipidomics results obtained in tears ($p < 0.05$).<br>• Significant alterations of AC levels were observed in tears from MS patients, suggesting a typical ACs signature. In particular, a significant ↑ of C5OH/C4DC ($p < 0.05$), C10:1 ($p < 0.001$), C8:1 ($p < 0.05$), and a significant ↓ of C12 ($p < 0.01$), C14:1 ($p < 0.001$) and C18:1OH ($p < 0.001$) were found in MS vs controls.<br>• There were ↑ tear levels of Ser p ($p < 0.01$), Asp ($p < 0.05$) and His ($p < 0.05$) in MS patients.
Kasakin et al, 2019	Plasma	22 RR MS/ 22 controls	LC-MS	• ↑ glutamic acid (glutamate toxicity mechanism) and ↓ BCAA (Leu + Ile) and C10:1 carnitine were observed in RR MS vs controls ($p < 0.05$). The ↓ in Leu e Ile could reflect the amino acid catabolism disorders during inflammation.<br>• Models based on a unique marker could not achieve the effectiveness of the generalized linear regression model based on all 4 biomarkers.
Sylvestre et al, 2019*	Plasma	28 RR MS/18 controls	NMR spectroscopy	• Brain fractional volumes (thalamus fractional volume, p=0.01 and brain parenchymal fraction, p=0.03) and 9 metabolites (arginine, isoleucine, citrate, serine, phenylalanine, methionine, asparagine, histidine, myo-inositol, $p < 0.05$) were significantly ↓ in RR MS vs controls.<br>• There was a significant correlation between arginine and T1 holes (p=0.0490)/white matter lesions (p=0.0358) and between methionine and T1 holes (p=0.009).<br>• Arginine, asparagine, serine and histidine were positively associated with performance on executive function test in RR MS but not controls.
Castro et al, 2019*	Plasma	1st *cohort*: 54 RR MS (27 normal/27 high BMI); 2nd *cohort*: 93 RR MS (42 normal/51 high BMI) and 50 controls (13 BMI/37 high BMI)	LC-MS	• The number of $CD14^+$ monocytes was significantly ↑ in MS patients within the high BMI group ($p < 0.05$).<br>• The ceramide species with differential abundance in MS patients with high BMI were not similarly elevated in controls with high BMI (possibly due to the process of myelin destruction).<br>• Monocytes exposed for 24 h to the ceramide levels detected in MS patients with high BMI showed ↑ proliferation and DNA methylation.<br>• There was a negative correlation between MRI brain volume measurements and monocyte cell counts (1st cohort: low BMI p=0.38; high BMI p=0.03; 2nd cohort: low BMI p=0.90; high BMI p=0.002). Similar trends were identified for disease activity and clinical disability.
**Metabolic differences among disease stages**				
Aasly et al, 1997	CSF	10 RR MS/10 CP MS /14 controls	NMR spectroscopy	• Significantly ↓ lactate (p=0.07) and glutamine (p=0.07) in MS patients compared to controls.<br>• No differences in any of the parameters measured in the CSF between CP and RR MS patients.<br>• Significant correlation between lactate and glutamine ($p < 0.0005$) in the MS group, which suggests a change in astrocytic metabolism.
Lutz et al, 2007	CSF	33 CIS (21 with active plaques [CIS group 1] and 12 without active plaques [CIS group 2])/10 controls	NMR spectroscopy	• BHIB levels significantly ↑ in CIS group 1 vs 2 (p=0.0144), maybe due to an increased uptake (or possibly leakage) from blood, perturbation of astrocytic gluconeogenesis or perturbation of microcirculation.<br>• Lactate significantly ↑ in CIS group 1 vs 2 (p=0.0433), perhaps due to anaerobic glycolysis in and/or around emerging plaques.<br>• Fructose levels ↑ in CIS group 2 (p=0.0161) and less in CIS group 1 (p=0.0142) vs controls, maybe due enhanced transport across the brain-CSF barrier.<br>• PCA unambiguously showed data clustering according to the three patients group investigated.

Regenold et al, 2008	CSF and serum	31 RR (22 remitted, 9 relapsed)/54 SP (37 stationary, 17 relapsed)/18 healthy controls	GS-MS	• Sorbitol ($p < 0.001$), fructose (p=0.017 and $p < 0.001$, respectively) and lactate (p=0.005 and $p < 0.001$, respectively) significantly ↑ in the CSF of RR and SP subjects vs controls.<br>• SP patients had consistently ↑ CSF concentrations of lactate and polyol pathway metabolites than RR patients (p=ns).<br>• CSF lactate concentration was positively correlated with sorbitol and fructose concentrations in RR (p=0.003 and p=0.104, respectively) and SP patients (p=0.002 and p=0.503, respectively), but negatively in healthy controls (p=0.038 and p=0.196, respectively).<br>• These findings supported an association between increased activity of extra-mitochondrial pathways of glucose metabolism and MS disease progression that begins during the RR stage of disease.
Tavazzi et al, 2011	Serum	170 MS (66.5% RR, 25.3% SP, 8.2% PP)/163 healthy controls	HPLC	• ↑ hypoxanthine, xanthine, uric acid, sum of circulating oxypurines (possible direct consequence of altered mitochondrial function), creatinine, uridine (indicator of tissue energy crisis), β-pseudouridine, MDA (indicator of ROS-mediated lipid peroxidation) and nitrite/nitrate in MS patients vs controls ($p < 0.001$).<br>• Significantly different values of creatinine, uric acid and sum of oxypurines in RR patients vs both SP and PP patients ($p < 0.01$).<br>• ↓ ascorbic acid in MS vs controls ($p < 0.001$), as a consequence of the increased oxidative/nitrosative stress occurring in MS.
Smolinska et al, 2012	CSF	For NMR, 26 MS/20 CIS. For GC-MS, 24 MS/14 CIS	NMR spectroscopy and GC-MS	• Both NMR and GC-MS held relevant information for discriminating CIS and MS groups.<br>• Metabolites having a relatively high contribution in the K-PLS-DA model were urea, glutamine, lactate, citrate and valine (already associated with MS, which gives a biological validation to the fusion of data model).
Reinke et al, 2014	CSF	11 RR-MS/3 SP-MS/1 CIS/17 non-MS (multiple diagnoses)	NMR spectroscopy	• Citrate, 3-hydroxybutyrate, phenylalanine, 2-hydroxyisovalerate and mannose significantly ↓ in MS vs non-MS ($p < 0.05$)<br>• Choline, myo-inositol and threonate significantly ↑ in MS vs non-MS ($p < 0.05$).<br>• By using HCA, 26 of the 32 specimens were correctly associated with their disease class (sensitivity 93%, specificity 71%).
Dickens et al, 2014	Serum	Set A (13 PP MS/38 SP MS/15 RR MS/10 controls); Set B (10 SP MS/6 RR MS/7 controls); Set C (10 SP MS/5 RR MS/7 controls)	NMR spectroscopy	• Significant separation between RR and SP groups (q^2=0.45). Phosphocholine (δ=3.23), some FAs (δ=0.88, 1.30, 5.35), an N-acetyl species (δ=2.03) and glucose were ↓ in SP vs RR MS, whereas β-hydroxybutyrate and other FAs (δ=1.19) were ↑.<br>• Models comparing PP vs RR and PP vs SP returned a nonpredictive q^2 value.<br>• When comparing each of the individual stage of MS vs controls, glucose (δ=3.25, 3.75, 3.91) and phosphocholine (δ=3.23) were both ↓.<br>• Lactate (δ=1.32), a broad singlet-like resonance assigned to N-acetyl species (δ=2.03) and some FAs were ↓ in RR MS and SP MS patients vs controls. A separate subset of FAs was ↑ in RR MS and SP MS patients vs controls. Lactate, N-acetyl species and some FAs were ↑ in PP MS patients vs controls.<br>• All Set B models were sensitive and specific at predicting group membership for Set C.
Senanayake et al, 2015	Serum	24 SP MS, 100 RR MS, 19 PP MS and 55 age-matched control subjects	Flow injection tandem mass spectrometry	• RR MS subjects having < 13 years disease duration had ↑ anti-inflammatory GTAs ($p < 0.05$) and normal levels of mitochondrial stress biomarkers (VLCFA-PtdEn) vs controls.<br>• SP MS subjects had statistically similar levels of GTAs, ↑ VLCFA-PtdEn ($p < 0.05$, elongation of FAs in peroxisomes) and ↑ PlsEtn ($p < 0.05$, due to ↑ availability of acetyl-CoA produced during peroxisomal β-oxidation) vs controls.<br>• RR MS subjects with ≥ 13 years disease duration exhibited metabolic profiles intermediate between short-duration RR MS and SP MS.<br>• PP MS patients exhibited metabolic profile distinct from RR MS and SP MS. The number of VLCFA species significantly ($p < 0.05$) ↑ in PP MS vs controls tended to be similar to the RR MS ≥ 13 years category.
Lazzarino et al, 2016*	Serum	518 MS (360 RR, 132 SP, 26 PP)/167 healthy controls	HPLC	• 9 metabolites (lactate, creatinine, and purine and pyrimidine compounds) were significantly ($p < 0.000001$) different in MS patients vs controls.<br>• A "Biomarker Score" (sum of the number of positive categories, i.e. number of metabolites having a concentration above the corresponding 95% percentile of controls) was calculated for each subject. MS patients had a mean Biomarker Score 10 times higher than controls ($p < 0.00001$). RR MS patients had Biomarker Scores lower than progressive MS patients ($p < 0.00001$), but there were no statistical differences among progressive forms of MS.

				• There was a high association between the Biomarker Score and increase in disability (EDSS), as with parameters of neurodegeneration detected by MRI (peripheral grey matter [$p < 0.01$], ventricular CSF [$p < 0.01$], NGMV [$p < 0.007$], NWMV [$p < 0.07$] and NBV [$p < 0.05$]).
Lim et al, 2017	Serum and CSF	1) 50 RR MS/20 SP MS/27 PP MS/49 controls;<br>2) 44 RR MS/15 SP MS;<br>3) 10 RR MS/20 SP MS/6 controls	GC-MS	• Kyn/Trp ratio significantly ↑ in MS patients vs healthy controls ($p < 0.0001$).<br>• KA ↑ in RR MS vs controls and progressive MS groups ($p < 0.0001$). KA significantly ↓ in progressive MS groups vs controls.<br>• Picolinic acid (neuroprotective KP metabolite) showed a similar trend to KA, being ↑ in RR MS but ↓ in PP MS ($p < 0.0001$).<br>• QA ↑ with disease severity and particularly ↑ in PP MS ($p < 0.0001$).<br>• The QA/KA ratio (indicative of excitotoxic potential) was ↑ in both PP MS and SP MS vs controls and RR MS ($p < 0.0001$). The QA/KA had the strongest correlation with EDSS (r=0.62, $p < 0.0001$).<br>• Kyn/Trp ratio significantly ↑ over time (p=0.029) in RR MS patients. Initially, induction of the KP may be beneficial due to immunomodulatory effect but chronic KP activation changes the excitotoxic balance.
Stoessel et al, 2018	Plasma	*Cross-sectional MS cohorts*: 33 PP MS/10 RR MS/33 controls; 15 PP MS participating in a *longitudinal cohort*.<br>PD study: 40 PD /20 controls	Modified hydrophilic interaction chromatograp hy combined with HRMS	• Levels of LysoPE(18:2) and LysoPC(20:0) were significantly ↓ in PP MS vs RR MS and PD (one-way ANOVA $p < 0.05$). Tyglicarnitine was significantly ↑ in PP MS vs RR MS (one-way ANOVA $p < 0.05$).<br>• Some metabolites were consistently downregulated in all diseases analysed, indicating a general signature of neurodegeneration, even if there were disease-specific differences, e.g. ↓ γ-Linolenic acid, L-Trp and LysoPC(20:0) in PP MS and ↑ of these analytes in RR MS and PD. Overall, the identified markers indicated a PP MS-specific plasma signature.<br>• In a longitudinal PP MS cohort, 18 of 20 determined metabolites showed no significant change over 24 months. However, there was a significant ↓ over time (12 vs 24 months p=0.0034; baseline vs 24 months p=0.0044) of LysoPC(20:0) which demonstrated a strong association with PP MS disease course.
Herman et al, 2018*	CSF	30 RR MS/16 SP SM/10 controls with non-inflammatory neurological diseases	LC-MS	• A combination of 11 variables (3 MRI variables: size of the spinal cord, size of the 3rd ventricle and number of T1 hypointense lesions; 6 proteins: galectin-9, monocyte chemoattractant protein-1, TGF-α, TNF-α, soluble CD40L and PDGF-AA; 2 metabolites: 20β-dihydrocortisol and indolepyruvate) was able to distinguish SP SM from RR MS better than any single measure ($p=8.5\times10^{-9}$).<br>• MBP (p=0.04), macrophage-derived chemokine (p=0.05) and 5,6-dihydroxyprostaglandin (p=0.05) predicted a worse disease progression in SP SM patients.
Herman et al, 2019*	CSF	30 RR MS/16 SP SM/10 controls with non-inflammatory, neurological diseases	HRMS	• Pathways affected in SP MS vs RR MS: aminoacyl-tRNA biosynthesis (p=0.00042); phenylalanine metabolism (p=0.0029); tryptophan metabolism (p=0.0039); valine, leucine and isoleucine biosynthesis (p=0.0055); pyrimidine metabolism (p=0.0083); nitrogen metabolism (p=0.015); valine, leucine and isoleucine degradation (p=0.016) and purine metabolism (p=0.035).<br>• KYNA (neuroprotective agent generated through deamination of KYN) was ↑ in SP MS vs RR MS (p=0.050).<br>• From the serotonin pathway, there were significantly ↑ levels of 5-HTP (↑ SP-C, p=0.016) and ↓ levels of 5-HIAA (↓ SP-C, p=0.101). Similar trends were found by comparing SP and RR-MS patients. 5-HIAA demonstrated associations with EDSS and size of the spinal cord ($p < 0.05$).
Murgia et al, 2020	CSF and serum	22 RR MS/12 PP MS	NMR spectroscopy, GC-MS and LC-MS	• The analysis of the serum samples identified PC aa C34:3 as the best lipid compound to classify the 2 groups ($p < 0.05$), while alpha-AAA was the most discriminant between amino acids and biogenic amines ($p < 0.05$).<br>• The analysis of the CSF samples identified PC ae C42:2 as the best lipid compound to classify the patients ($p < 0.05$), while between amino acids and biogenic amines the most discriminant was histidine ($p < 0.05$).<br>• Common altered pathways between CSF and serum were oxidative stress and arginine metabolism.
Yeo et al, 2020	Serum	*Prospective cohort*: 31 RR MS/28 SP MS; *cohort to determine the effect of long-term storage*: 30 RR MS/50 SP MS	NMR spectroscopy	• OPLS-DA models showed excellent separation between RR MS and SP MS (predictive accuracy 91± 3.0%). Lipoproteins (VIP score 2.15, 2.52, 3.10, 4.29), choline (VIP score 2.38, 3.69), and 3-hydroxybutyrate (VIP 2.07) were ↓ in SP MS vs RR MS, while glucose (VIP score 1.50) and N-acetylated glycoproteins/glycolipids (VIP score 1.48) were ↑.<br>• No strong correlation was detected among discriminatory metabolites and demographic or clinical variables.<br>• The well-validated OPLS-DA models were able to predict the diagnosis of samples from the freeze-thaw, 120 min and 240 min protocols. The 7 discriminatory metabolites identified by the well-validated model remained discriminatory with variations in standing time, while most were still able to distinguish RR MS vs SP MS despite an additional freeze-thaw.

**Metabolic differences between MS and other inflammatory demyelinating diseases**				
Moussallieh et al, 2014	Serum	47 MS/44 NMO (22 anti AQP4-antibodies positive and 22 seronegative)/42 controls	NMR spectroscopy	• Scyllo-inositol in MS patients had a substantial 2.4-fold ↑ vs NMO ($p < 0.0001$) and a significant 2.6-fold ↑ vs controls ($p < 0.0001$), indicating an increased release or decreased uptake by the brain. A cut-off value of 190.3 µmol/L was determined for discriminating MS from NMO patients.<br>• Acetate in NMO patients had a substantial 1.8-fold ↑ vs MS ($p < 0.0001$) and a significant 3.4-fold ↑ vs controls ($p < 0.0001$), perhaps due to impairment of acetate uptake by astrocyte. A cut-off value of 18.5 µmol/L was determined for discriminating NMO from MS patients.<br>• Scyllo-inositol and acetate concentrations did not specifically correlate with the anti-AQP4 status, so there was no distinction between anti-AQP4 seropositive and seronegative patients in the models.
Park et al, 2016	CSF	54 MS /49 NMOSD/30 ITM/12 normal controls	GC-MS	• 1-monopalmitin (p=8.01E-06), 1-monostearin (p=4.37E-06) and glycolic acid (p=4.52E-02) significantly ↑ and glycine (p=3.67E-02), inosine (p=8.22E-03), threose (p=2.08E-06) and butane-2,3-diol (p=1.07E-03) significantly ↓ in the disease group, possibly due to cellular toxicity in common across different types of autoimmune inflammatory disorders of the CNS.<br>• Multiple metabolites in coordinated regression with clinical characteristics, EDSS, oligoclonal bands and proteins successfully discriminated the 4 different immune statuses (control-others AUC 0.991; MS-others AUC 0.904; NMOSD-others AUC 0.869; ITM-others AUC 0.842).<br>• 14 metabolites with gradual ↑ in MS (control < remission < relapse) were linked to FA (possible dysfunction in β-oxidation) and arginine-proline metabolisms in MS-relapse. A similar dysregulation in FA metabolism was seen in the relapse stage of NMOSD.
Gebregiworgis et al, 2016	Urine	8 RR MS, 9 anti-AQP4 antibodies positive NMOSD patients and 7 healthy controls	NMR spectroscopy	• 26 metabolites were differentially altered in the urine samples of healthy controls and MS patients, from metabolic pathways associated with energy metabolism, FA synthesis and gut microflora. Especially, the set of metabolites including creatinine (p=0.00042), 3-hydroxyisovalerae (p=0.032) and oxaloacetate (p=0.05) discriminated between healthy controls and MS patients.<br>• Creatinine (p=0.0000004), 3-hydroxybutyrate (p=0.029), methylmalonate (p=0.026) and oxaloacetate (p=0.027) had the potential of differentiating between healthy controls and NMOSD patients.<br>• Urine from MS patients exhibited a statistically distinct metabolic signature from NMOSD patients, with a total of 20 metabolites differentially altered. The identified metabolites were amino acids and their derivatives, tricarboxylic acid cycle intermediates, choline containing compounds and metabolites from the gut microflora. The set of metabolites including creatinine (p=0.015), 3-hydroxybutirate (p=0.008), 3-hydroxyisovalerate (p=0.000084) and methylmalonate (p=0.00011) discriminated between MS and NMOSD patients.
Kim et al, 2017	CSF	50 MS/57 NMOSD/17 healthy controls	NMR spectroscopy	• 2-hydroxybutyrate, acetone, formate and pyroglutamate ↑ while glucose and acetate ↓ in MS ($p < 0.003$) and NMOSD ($p < 0.001$) vs healthy controls. Citrate ↓ only in MS (p=0.003) and lactate ↑ only in NMOSD ($p < 0.001$).<br>• 2-hydroxybutyrate, acetone and formate ↑ and acetate and glucose ↓ in both relapse and remission of MS and NMOSD ($p < 0.01$).<br>• The level of pyroglutamate tend to ↑ in both relapse (p=0.006) and remission ($p < 0.001$) of NMOSD.<br>• Citrate ↓ in both relapse ($p < 0.001$) and remission (p=0.004) of MS and lactate ↑ in both relapse ($p < 0.001$) and remission (p=0.002) of NMOSD.<br>• Isoleucine and valine ↓ in MS relapse vs remission (p=0.003), whereas iso-butyrate ↓ in NMOSD relapse vs remission (p=0.002).
Jurynczyk et al, 2017	Plasma	34 RR MS, 54 AQP4 antibodies positive NMOSD and 20 MOG-Ab disease	NMR spectroscopy + lipoprotein profiling	Relative to the other diseases ($p < 0.001$):<br>• The AQP4 NMOSD metabolic profile was characterized by ↓ scyllo-inositol and small HDL particles along with ↑ large LDL particles.<br>• RR MS plasma exhibited ↑ histidine and glucose, along with ↓ lactate, alanine and large HDL particles.<br>• MOG-Ab disease plasma was defined by ↑ formate and leucine coupled with ↓ myo-inositol.
**Monitoring of biological response to treatment**				
Kallaur et al, 2016	Plasma	212 MS /249 healthy controls	ELISA, chemiluminescence	• IL-10 (p=0.023), TNF-α (p=0.001), INF-γ (p=0.032), advanced oxidation protein products (p=0.002) and NO ($p < 0.001$ metabolites significantly ↑ and IL-4 (p=0.025) significantly ↓ in patients with an EDSS ≥ 3 vs those with EDSS < 3.<br>• ↑ INF-γ was associated with higher pyramidal symptoms (p=0.041) and ↑ IL-6 with sensitive symptoms (p=0.029).

				• ↑ carbonyl protein (p=0.012) and IL-10 (p=0.036) but ↓ albumin levels (p=0.017) predicted cerebellar symptoms (different mechanisms may underpin the impairment of functional systems).<br>• Treatment with IFN-β (p=0.001) and glatiramer acetate (p=0.01) significantly ↓ TNF-α.
Lorefice et al, 2019	Plasma	21 MS starting IFNβ (16 responders/5 non-responders)/16 controls	NMR spectroscopy	• Acetoacetate, acetone, 3-OH-butyrate, glutamate and methylmalonate significantly ↓ during treatment, whereas tryptophan ↑ ($p < 0.005$).<br>• In baseline samples, lactate, acetone and 3-OH-butyrate were significantly ↑ in non-responders, whereas lysine and glucose levels were significantly ↑ in responders (p=0.01).
Waddington et al, 2020	Serum and plasma	82 MS patients (52 ADA- and 30 ADA +)	ELISA and NMR spectroscopy	• Seven lipids were significantly ↑ in patients who went on to develop ADA: triglycerides in medium HDL particles ($p < 0.03$), ApoB/ApoA1 ($p < 0.03$), cholesterol esters in medium VLDL particles (p < 0.01), free cholesterol in chylomicrons and very large VLDL particles ($p < 0.0003$), remnant cholesterol ($p < 0.01$), VLDL diameter ($p < 0.01$), ratio of triglycerides to phosphoglycerides ($p < 0.03$).<br>• Some of the metabolite and parameters increases induced by IFNβ treatment in ADA^- were inhibited in ADA^+ patients: concentration of very small VLDL particles, phospholipids in very small VLDL particles, ratio of triglycerides to phosphoglycerides, ratio of saturated FA to total FA, triglycerides in small VLDL particles, total cholines and total phosphoglycerides ($p < 0.01$).<br>• Other metabolites and parameters were more suppressed in ADA^+ vs ADA^- individuals: ratio of polyunsaturated FA to total FA, estimated degree of saturation and serum total triglycerides ($p < 0.05$).<br>• Before exposure to IFNβ, plasma membrane cholesterol was ↑ and glycosphingolipids ↓ in $CD4^+$ isolated from ADA^+ patients ($p < 0.05$, serum lipids may contribute to ADA development by altering immune-cell lipid rafts).
Signoriello et al, 2020	Serum	28 MS/27 controls	ELISA for PTX-3 levels and NMR spectroscopy	• PTX-3 levels were ↑ in MS vs controls (p=0.03). No difference in PTX-3 levels were found in patients with high lesion load, spinal cord lesions and between active or inactive patients.<br>• Metabolomic evaluation shows ↑ lactate and ↓ isoleucine, hydroxyproline, phenylalanine, 2-hydroxybutyrate, three oxidative phosphorylation markers, valine, tyrosine and tryptophan in MS vs controls.<br>• During therapy, PTX-3 levels were significant ↓ at 6 months and one year of treatment (p=0.001). ↓ in PTX-3 level was higher in fully-responders vs non-responders.<br>• There was a higher ↓ in lactate, tyrosine and hypoxanthine and a ↑ in hydroxyproline, ADP, three oxidative phosphorylation markers, citrulline, ornithine and tryptophan in fully-responders vs non-responders, approaching the metabolic profile of controls.
